# Supplementary material for: Epigenetic conditioning improves sequence-based modeling of gene regulation across cell types and alleles
Source: bioRxiv. 2026 Jun 7:2026.06.02.729723. Preprint. [Version 1] doi: 10.64898/2026.06.02.729723 (PMC13252088; doi:10.64898/2026.06.02.729723)
Supplement: Supplement 2 [file NIHPP2026.06.02.729723v1-supplement-2.pdf]

bioRxiv preprint doi: https://doi.org/10.64898/2026.06.02.729723; this version posted June 7, 2026. The copyright holder for this preprint (which was not certified by peer review) is the author/funder, who has granted bioRxiv a license to display the preprint in perpetuity. It is made available under a CC-BY 4.0 International license.

Supplementary Material

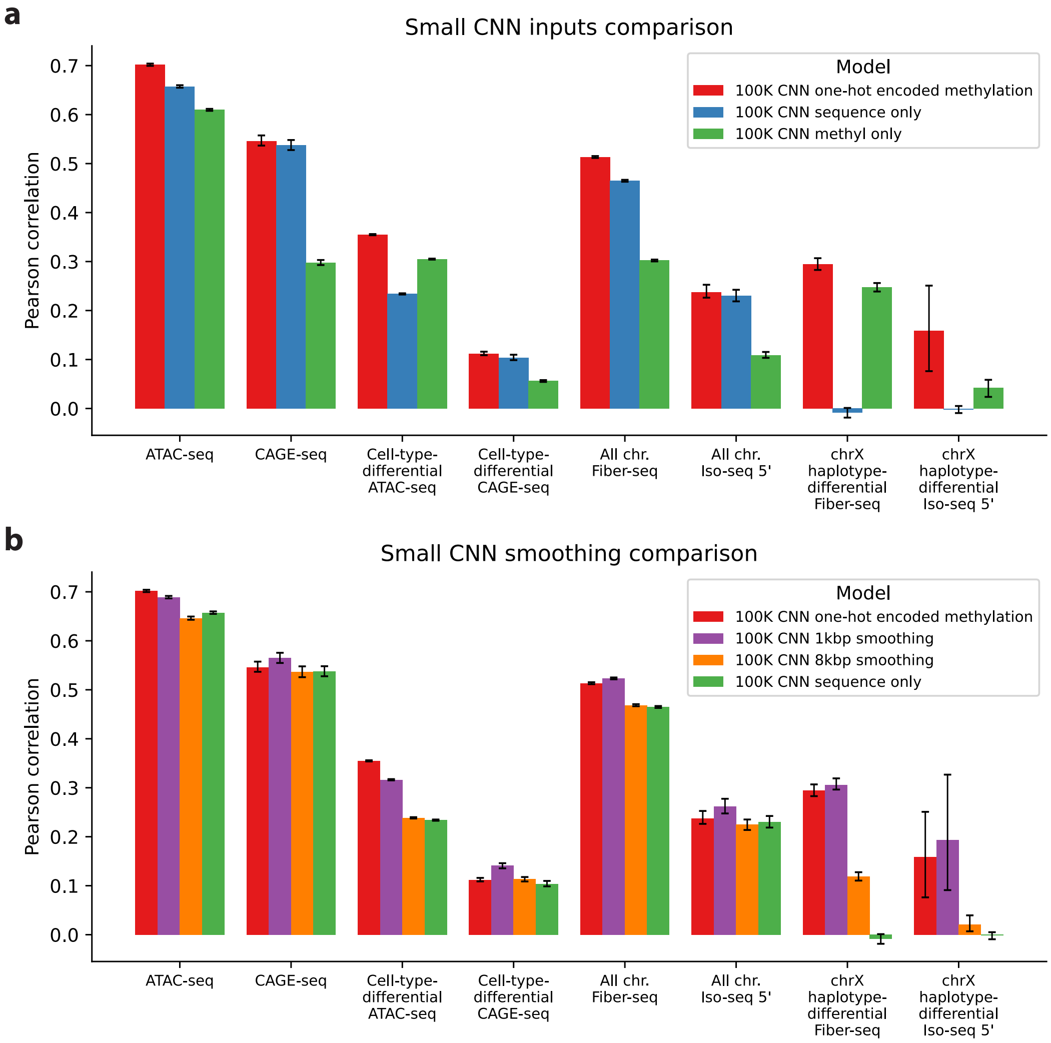

Supplementary Fig. 1 Small CNN encoder ablations. **a**, Performance comparison of small CNN models with sequence and methylation input, just sequence, and just methylation. 95% confidence intervals from 100 bootstrap resamples (N = full test set subset size, with replacement) are shown. **b**, As in a but for small CNN models with different levels of input methylation smoothing.

bioRxiv preprint doi: https://doi.org/10.64898/2026.06.02.729723; this version posted June 7, 2026. The copyright holder for this preprint (which was not certified by peer review) is the author/funder, who has granted bioRxiv a license to display the preprint in perpetuity. It is made available under a CC-BY 4.0 International license.

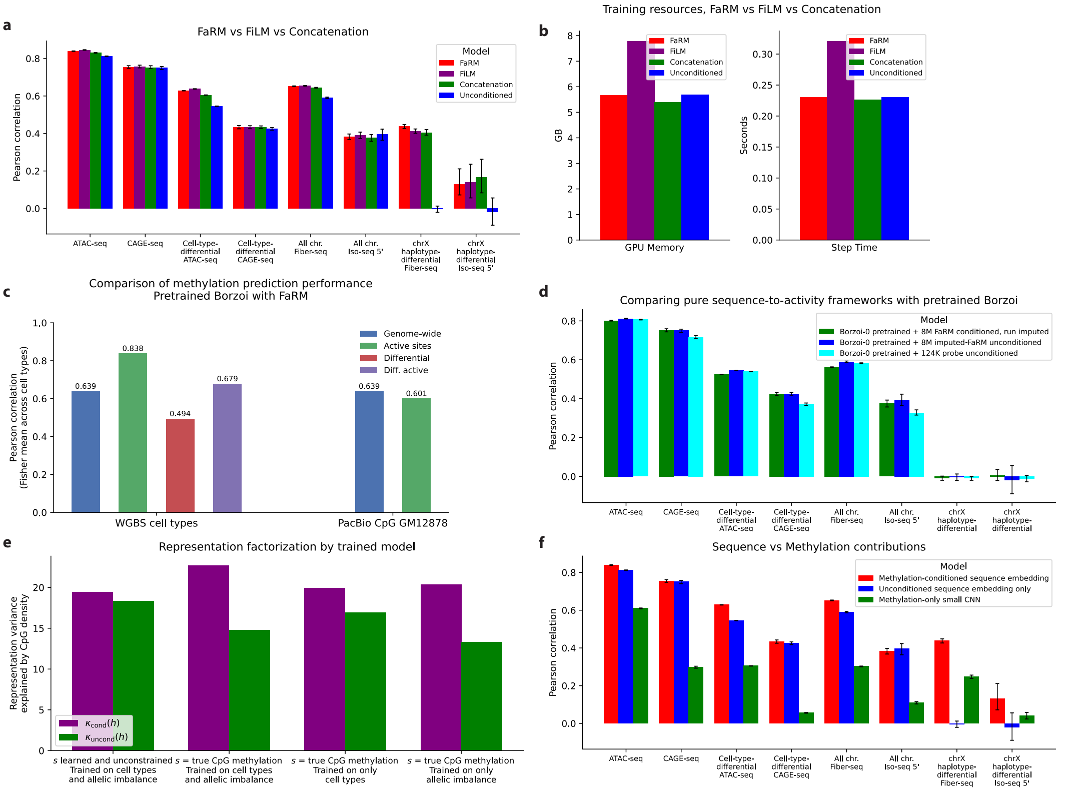

Supplementary Fig. 2 FaRM performance extended. **a**, Prediction performance comparisons of FaRM conditioning, FiLM conditioning, simple concatenation of the conditioning representation, and the unconditioned MethylSeqNet. 95% confidence intervals from 100 bootstrap resamples (N = full test set subset size, with replacement) are shown. **b**, GPU memory and training step time comparisons of FaRM conditioning, FiLM conditioning, simple concatenation of the conditioning vector, and the unconditioned MethylSeqNet. **c**, Imputed methylation performance comparison across cell types and data types. **d**, As in **a** but comparing FaRM output head run with imputed conditioning, an unconditioned FaRM output head, and a linear probe on the Borzoi embeddings. **e**, Comparison of representation variance explained by CpG density for conditional and unconditional representations across different MethylSeqNet configurations. **f**, As in **a** but comparing MethylSeqNet with pretrained Borzoi, both conditioned and unconditioned, and a methylation-only CNN model.

bioRxiv preprint doi: https://doi.org/10.64898/2026.06.02.729723; this version posted June 7, 2026. The copyright holder for this preprint (which was not certified by peer review) is the author/funder, who has granted bioRxiv a license to display the preprint in perpetuity. It is made available under a CC-BY 4.0 International license.

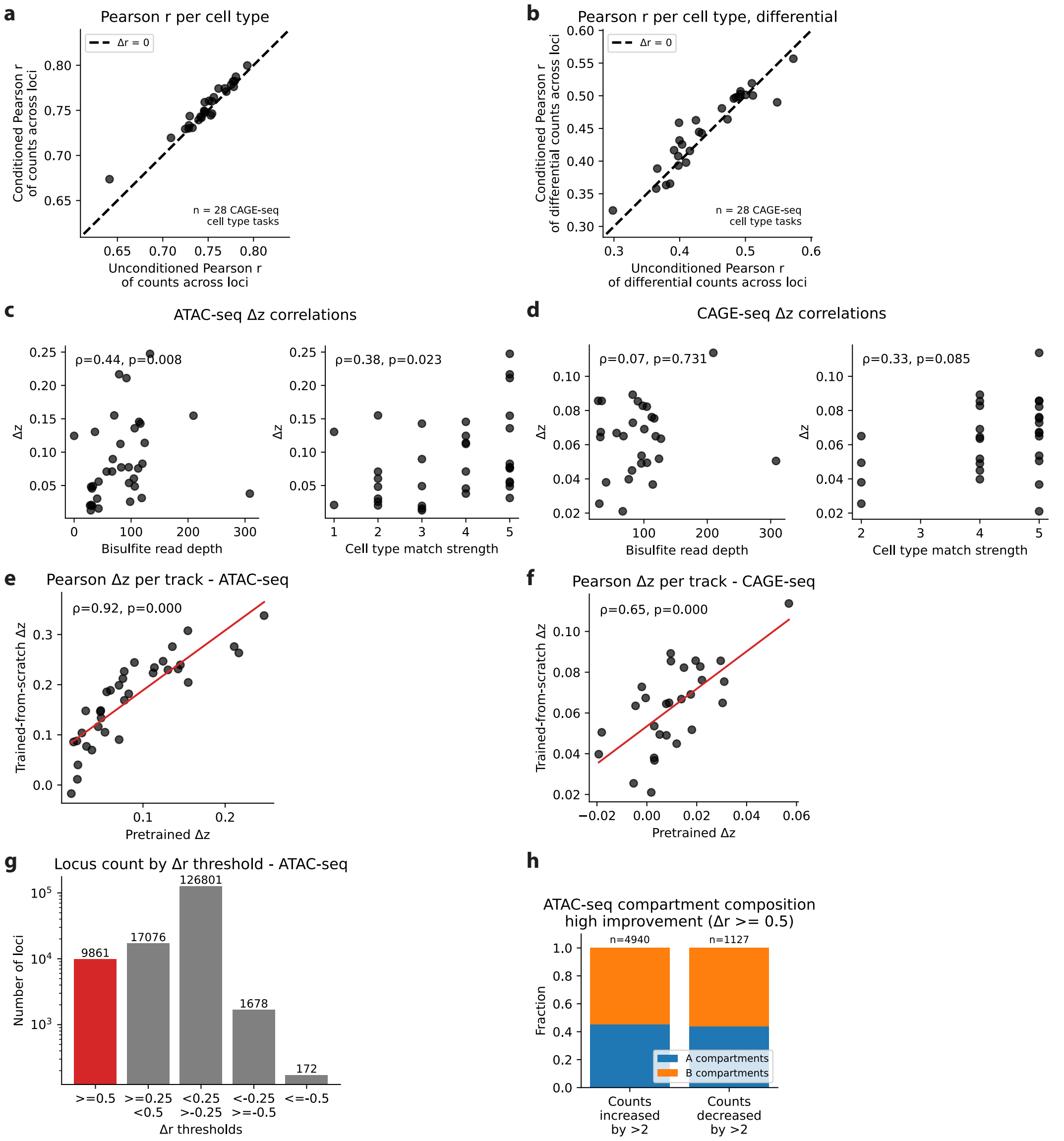

Supplementary Fig. 3 Cell type taskwise improvements. **a**, Scatterplot comparison of MethylSeqNet conditioned vs unconditioned prediction-measurement Pearson correlation for CAGE-seq test set by cell type. Each point represents one cell type/task, and the dashed line indicates equal performance. **b**, As in **a**, but for cell-type-differential Pearson correlation calculated from the difference from mean activity across cell types for the input sequence (Methods). **c**, ATAC-seq Fisher-z-transformed Pearson (Δz) conditioned-unconditioned by cell type correlated to WGBS read depth and to WGBS:ATAC-seq cell type match quality, showing Spearman correlation. **d**, As in **c**, but for CAGE-seq. **e**, ATAC-seq Fisher-z-transformed Pearson (Δz) conditioned-unconditioned by cell type for two MethylSeqNet configurations: a trained-from-scratch Basenji2 sequence encoder compared with the pretrained Borzoi encoder used for main-text analysis. **f**, As in **e**, but for CAGE-seq. **g**, Distribution of peak loci in terms of ATAC-seq per-locus cross-cell-type Pearson correlation change from Fig. 2c. **h**, Loci highlighted in **g** with per-locus cross-cell-type Pearson correlation increased by >0.5, assessed for all cell types with matched A and B compartment data from [31] and subset to cell types with >2 counts shift from unconditional to conditional predictions, as shown in Fig. 2d. Sites are localized into annotated A and B compartments for higher-activity-with-conditioning and lower-activity-with-conditioning peaks.

bioRxiv preprint doi: https://doi.org/10.64898/2026.06.02.729723; this version posted June 7, 2026. The copyright holder for this preprint (which was not certified by peer review) is the author/funder, who has granted bioRxiv a license to display the preprint in perpetuity. It is made available under a CC-BY 4.0 International license.

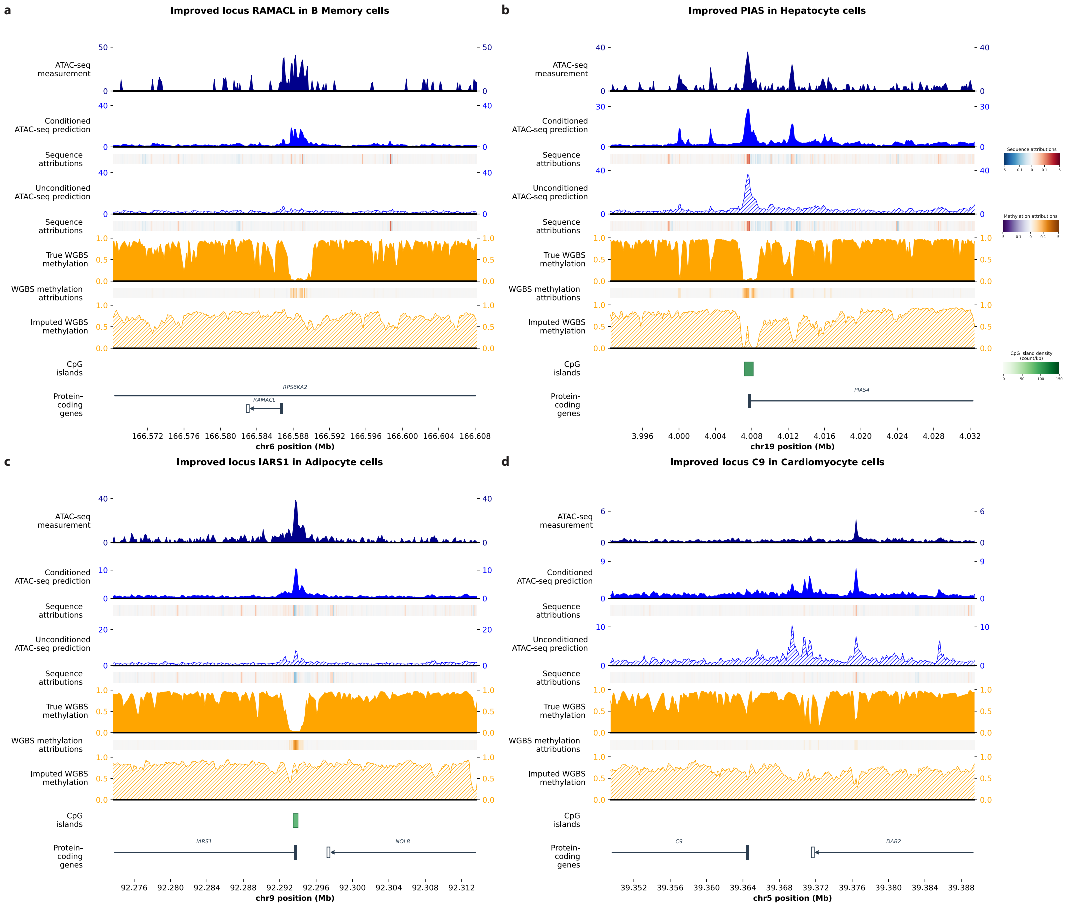

Supplementary Fig. 4 Visualizations of improved activity prediction at several loci. a,b,c,d, Examples of loci where MethylSeqNet improves accessibility prediction: *RAMACL* in B memory cells, *PIAS4* in Hepatocytes, *IARS1* in Adipocytes, and *C9* in Cardiomyocytes. We visualize genomic tracks for accessibility measurements, conditioned and unconditioned predictions from MethylSeqNet along with their respective sequence attributions calculated using integrated gradients, WGBS methylation measurements, MethylSeqNet imputed WGBS methylation, methylation attributions calculated using integrated gradients, and CpG island and gene annotations.

bioRxiv preprint doi: https://doi.org/10.64898/2026.06.02.729723; this version posted June 7, 2026. The copyright holder for this preprint (which was not certified by peer review) is the author/funder, who has granted bioRxiv a license to display the preprint in perpetuity. It is made available under a CC-BY 4.0 International license.

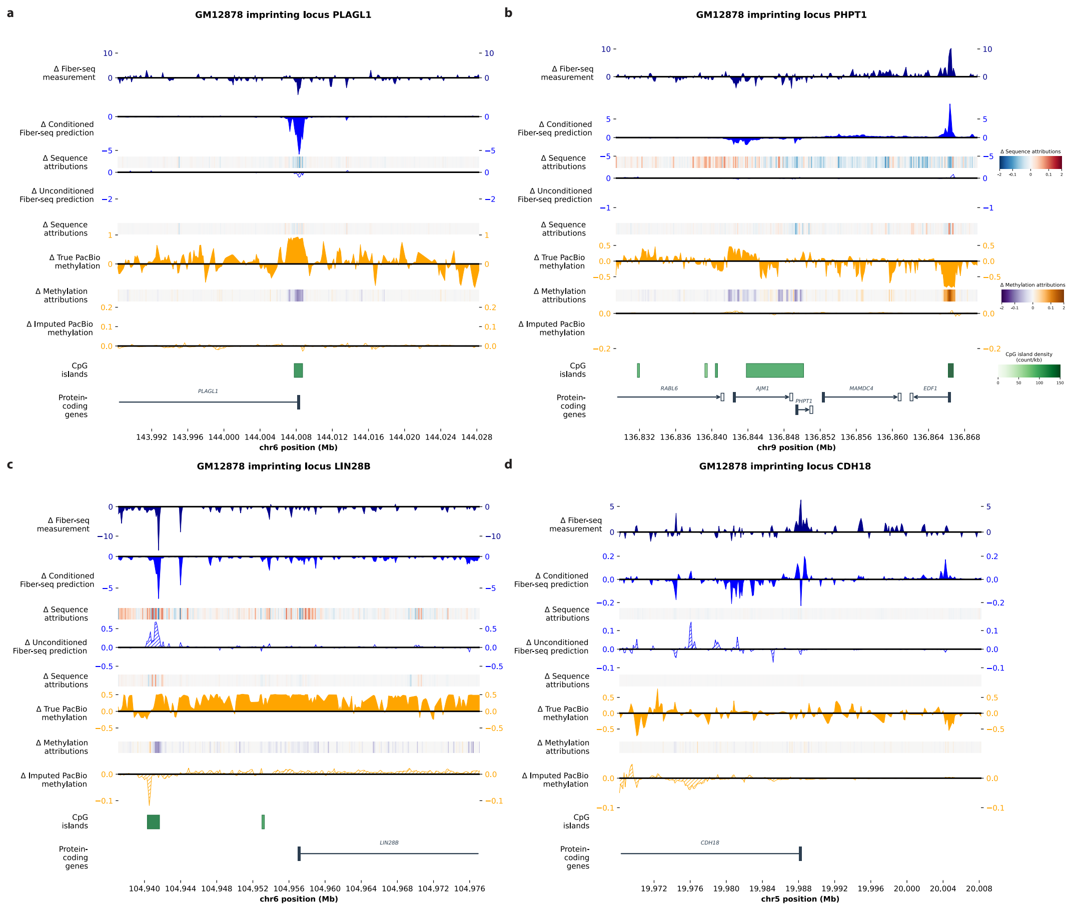

Supplementary Fig. 5 Visualizations of improved differential activity prediction at several imprinting loci. a,**b**,**c**,**d**, Examples of imprinting loci where MethylSeqNet improves haplotype-differential accessibility prediction: *PLAGL1*, *PHPT1*, *LIN28B*, and *CDH18*, all in GM12878 lymphoblastoid cells. We visualize haplotype-differential genomic tracks for accessibility measurements, conditioned and unconditioned predictions from MethylSeqNet along with their respective sequence attributions calculated using integrated gradients, PacBio methylation measurements, MethylSeqNet imputed PacBio methylation, methylation attributions calculated using integrated gradients, and CpG island and gene annotations.

bioRxiv preprint doi: https://doi.org/10.64898/2026.06.02.729723; this version posted June 7, 2026. The copyright holder for this preprint (which was not certified by peer review) is the author/funder, who has granted bioRxiv a license to display the preprint in perpetuity. It is made available under a CC-BY 4.0 International license.

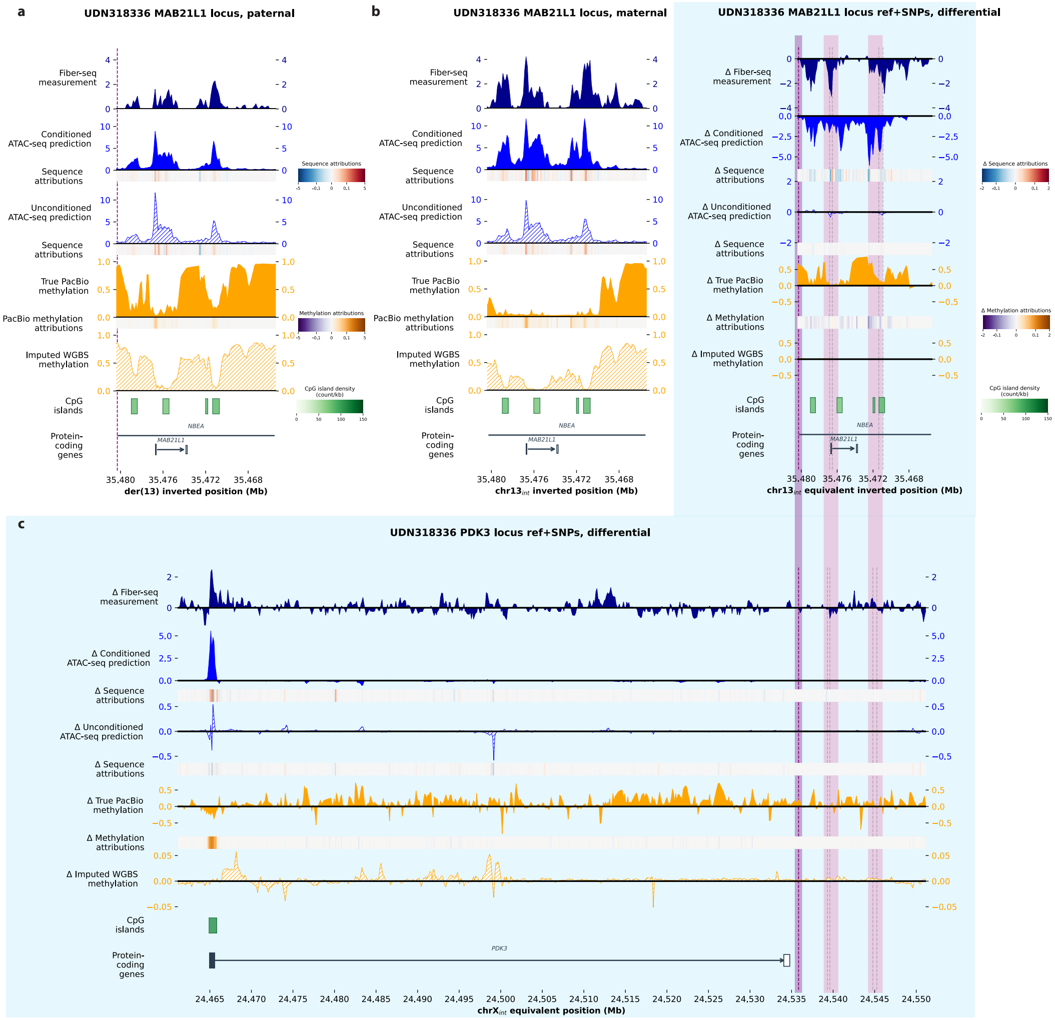

Supplementary Fig. 6 Undiagnosed disease patient loci. a, **b**, The *MAB21L1* locus shows large activity differences for paternal and maternal haplotypes. We visualize genomic tracks for accessibility measurements, conditioned and unconditioned activity predictions from MethylSeqNet along with their respective sequence attributions calculated using integrated gradients, PacBio methylation measurements, MethylSeqNet imputed methylation, methylation attributions calculated using integrated gradients, and CpG island and gene annotations. **c**, The *PDK3* and *MAB21L1* loci haplotype-differential activity predicted without inclusion of the large structural variant. Blue background indicates that the reference sequence with haplotype-specific SNPs was used, rather than the structural variant sequences. We visualize haplotype-differential genomic tracks for accessibility measurements, conditioned and unconditioned ATAC-seq predictions from MethylSeqNet's neuron task along with their respective sequence attributions calculated using integrated gradients, PacBio methylation measurements, MethylSeqNet imputed WGBS methylation for neurons, methylation attributions calculated using integrated gradients, and CpG island and gene annotations. The fusion site (purple) and relevant promoters and enhancers (pink) are highlighted.

bioRxiv preprint doi: https://doi.org/10.64898/2026.06.02.729723; this version posted June 7, 2026. The copyright holder for this preprint (which was not certified by peer review) is the author/funder, who has granted bioRxiv a license to display the preprint in perpetuity. It is made available under a CC-BY 4.0 International license.

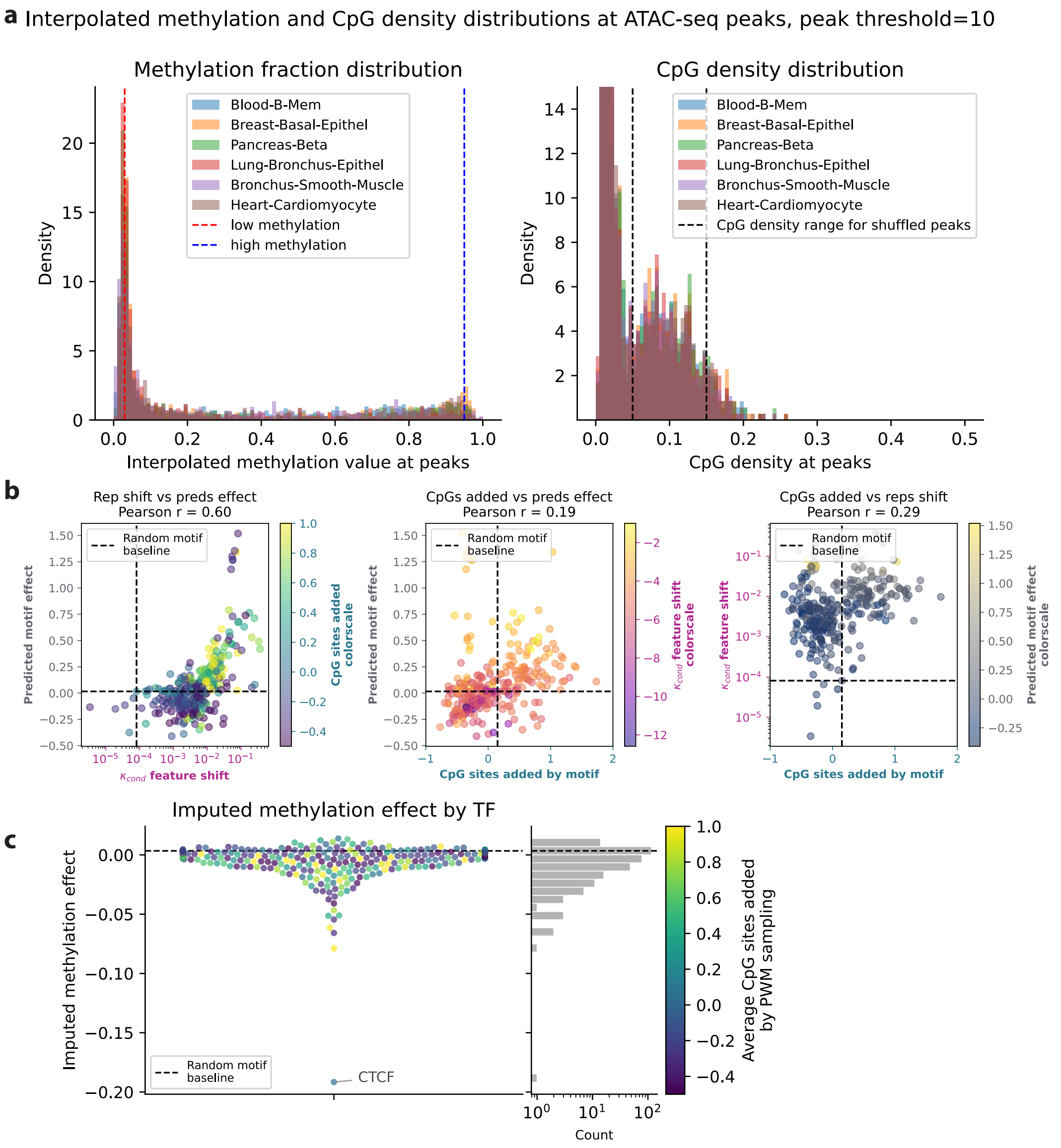

Supplementary Fig. 7 Further characterization of in silico motif insertion. a, Distributions of the accessibility peak methylation fraction distribution for WGBS when interpolated between CpG sites (left), and of the accessibility peak CpG density (right), for representative cell types. Dashed lines indicate values and ranges used for motif insertion. **b**, Scatterplots illustrate pairwise comparisons between three variables: chromatin accessibility TF motif effect, conditional feature shift, and average number of CpG sites added by a sampled position-weight matrix for that TF. The random motif baseline indicates values computed for a uniform random sampled motif. **c**, Swarmplot and corresponding histogram of the TF motif effect on imputed methylation predictions, showing CTCF as a strong outlier. Points are colored by the average number of CpG sites added by a sampled position-weight matrix for that TF.

bioRxiv preprint doi: https://doi.org/10.64898/2026.06.02.729723; this version posted June 7, 2026. The copyright holder for this preprint (which was not certified by peer review) is the author/funder, who has granted bioRxiv a license to display the preprint in perpetuity. It is made available under a CC-BY 4.0 International license.

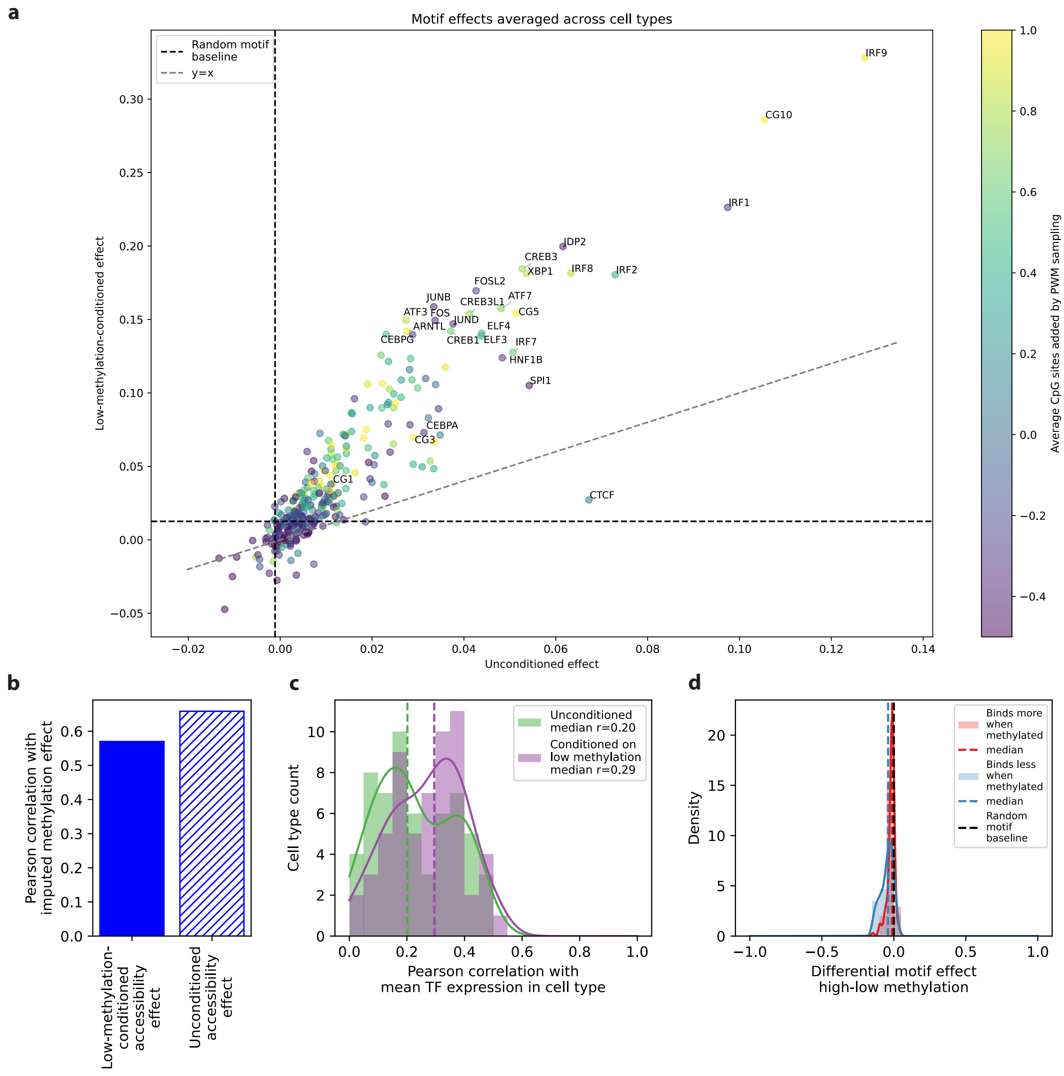

Supplementary Fig. 8 CAGE-seq TF motif effects from motif insertion analysis. a, Scatterplot of low-methylation-conditioned CAGE TF motif effect against unconditioned CAGE TF motif effect for all 301 TFs studied, averaged across all cell types. Points are colored by the average number of CpG sites added by a sampled position-weight matrix for that TF (the scale is truncated to exclude the all-CpG controls CG10, CG5, etc.), and several of the highest-effect TFs are highlighted. Black horizontal and vertical dashed lines indicate the calculated motif effect of a uniform random motif of length 10 for low methylation and unconditioned cases, and the gray dashed line indicates $y = x$. **b**, Correlation of the CAGE TF motif effects calculated using imputed methylation with those calculated using low methylation (left, solid) and no methylation conditioning (right, striped). **c**, Histograms of Pearson correlations per cell type between expression TF motif effect and corresponding TF expression in each cell type, plotted for low-methylation-conditioned vs unconditioned predictions. **d**, Histograms of differential CAGE TF motif effects (calculated as high methylation effect minus low methylation effect) for TFs known to bind more with methylation (red) vs those known to bind less (blue).

bioRxiv preprint doi: https://doi.org/10.64898/2026.06.02.729723; this version posted June 7, 2026. The copyright holder for this preprint (which was not certified by peer review) is the author/funder, who has granted bioRxiv a license to display the preprint in perpetuity. It is made available under a CC-BY 4.0 International license.

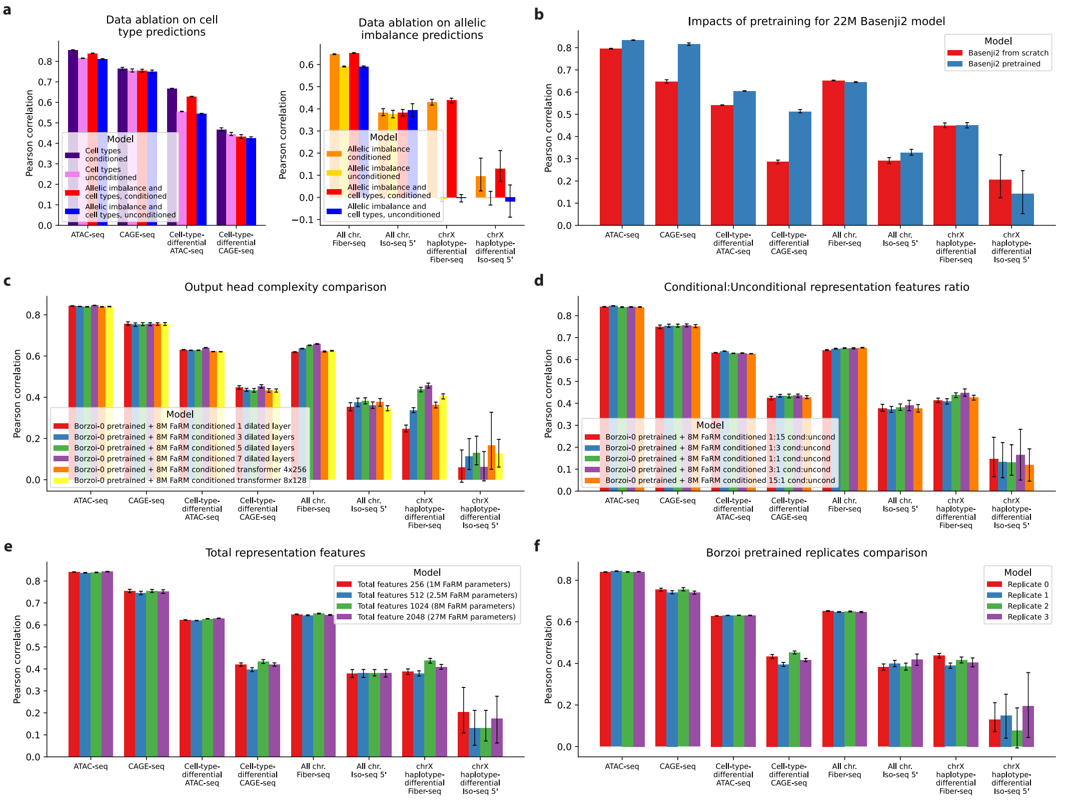

Supplementary Fig. 9 Performance comparisons of model ablations. a, Performance comparison from training conditioned and unconditioned MethylSeqNet on cell type and allelic imbalance data ablations, for the assay-relevant subsets of the data and output tasks. 95% confidence intervals from 100 bootstrap resamples (N = full test set subset size, with replacement) are shown. **b**, As in **a** but comparing Basenji2 trained from scratch vs pretrained in a MethylSeqNet architecture for various subsets of the data and output tasks, including both standard activity prediction and differential activity prediction between two sequences. **c**, As in **b** but comparing output (prediction) head architectures with increasing numbers of dilated convolution layers and two transformer layer configurations. **d**, As in **b** but comparing different ratios of conditional to unconditional features. **e**, As in **b** but comparing total representation features (conditional and unconditional). **f**, As in **b** but comparing the 4 pretrained Borzoi replicates.
